# *Pou5f1* and *Nanog* Are Reliable Germ Cell-Specific Genes in Gonad of a Protogynous Hermaphroditic Fish, Orange-Spotted Grouper (*Epinephelus coioides*)

**DOI:** 10.3390/genes13010079

**Published:** 2021-12-29

**Authors:** Chaoyue Zhong, Meifeng Liu, Yuhao Tao, Xi Wu, Yang Yang, Tong Wang, Zining Meng, Hongyan Xu, Xiaochun Liu

**Affiliations:** 1 State Key Laboratory of Biocontrol, Guangdong Province Key Laboratory for Improved Variety Reproduction of Aquatic Economic Animals, Institute of Aquatic Economic Animals, School of Life Sciences, Sun Yat-sen University, Guangzhou 510275, China; zhongchy9@mail2.sysu.edu.cn (C.Z.); liumeifeng1994@163.com (M.L.); taoyh3@mail2.sysu.edu.cn (Y.T.); wuxi577@126.com (X.W.); yyang0103@163.com (Y.Y.); sysuwangtong@163.com (T.W.); mengzn@mail.sysu.edu.cn (Z.M.); 2 Southern Marine Science and Engineering Guangdong Laboratory (Zhuhai), Zhuhai 519080, China; 3 Key Laboratory of Freshwater Fish Reproduction and Development, Ministry of Education, Key Laboratory of Aquatic Sciences of Chongqing, College of Fisheries, Southwest University, Chongqing 402460, China

**Keywords:** *Pou5f1*, *Nanog*, germ cell, protogynous hermaphroditic fish, orange-spotted grouper

## Abstract

Pluripotency markers Pou5f1 and Nanog are core transcription factors regulating early embryonic development and maintaining the pluripotency and self-renewal of stem cells. Pou5f1 and Nanog also play important roles in germ cell development and gametogenesis. In this study, *Pou5f1* (*EcPou5f1*) and *Nanog* (*EcNanog*) were cloned from orange-spotted grouper, *Epinephelus coioides*. The full-length cDNAs of *EcPou5f1* and *EcNanog* were 2790 and 1820 bp, and encoded 475 and 432 amino acids, respectively. *EcPou5f1* exhibited a specific expression in gonads, whereas *EcNanog* was expressed highly in gonads and weakly in some somatic tissues. In situ hybridization analyses showed that the mRNA signals of *EcNanog* and *EcPou5f1* were exclusively restricted to germ cells in gonads. Likewise, immunohistofluorescence staining revealed that EcNanog protein was limited to germ cells. Moreover, both *EcPou5f1* and *EcNanog* mRNAs were discovered to be co-localized with *Vasa* mRNA, a well-known germ cell maker, in male and female germ cells. These results implied that *EcPou5f1* and *EcNanog* could be also regarded as reliable germ cell marker genes. Therefore, the findings of this study would pave the way for elucidating the mechanism whereby *EcPou5f1* and *EcNanog* regulate germ cell development and gametogenesis in grouper fish, and even in other protogynous hermaphroditic species.

## 1. Introduction

Germ cell development is indispensable for animal reproduction and fertility. The complex process from the formation of primordial germ cells (PGCs) to the differentiation into gametes is strictly regulated by many factors, such as hormones and reproduction-related genes [1,2,3]. Germ cell marker genes can be used for exploring PGCs formation and migration, germ cell development, and the mechanism behind gametogenesis. In teleost fish, some germ cell-specific genes have been characterized, including PGC-specific gene *Nanos3* [4], mitotic germ cell-specific gene *Ly75* [5], spermatogonium-specific gene *Plzf* [6,7], oocyte-specific gene *Slbp2* [8], as well as the well-known germline-specific genes *Vasa*, *Dazl* and *Piwi* [9,10].

Pou5f1 (also known as Oct4) and Nanog are closely related to each other and characterized as core transcription factors essential for embryogenesis and the maintenance of cell pluripotency [11,12,13]. Pou5f1 protein is a class V POU domain transcription factor containing a POU domain that consists of POU specific domain (POUs), POU homeodomain (POUhD), and a linker between them [14]. The POU domain of Pou5f1 can regulate the transcription of its target genes possessing an octamer sequence motif of ATGC(A/T)AAT [15]. Most interestingly, the combined actions of *Pou5f1*, *Sox2*, *Klf4*, and *C-myc* are capable of reprogramming somatic cells into induced pluripotent stem cells (IPSCs) [16]. Nanog protein is a DNA binding homeobox transcription factor with a homeodomain (HD) [12,17]. The existence of OCT-SOX enhancer in *Nanog* promoter indicates that Pou5f1 and Sox2 are direct modulators of *Nanog* transcript [18]. Moreover, the protein complex formed by Nanog and Pou5f1 can influence the activity of at least 300 target genes [19]. In human (*Homo sapiens*) and mouse (*Mus musculus*), *Pou5f1* and *Nanog* are mainly expressed in multipotent stem cells, such as embryonic stem cells (ESCs), IPSCs, PGCs, spermatogonial stem cells, and oogonial stem cells [16,20,21]. In fish, such as zebrafish (*Danio rerio*), medaka (*Oryzias latipes*), and Japanese flounder (*Paralichthys olivaceus*), *Pou5f1* and *Nanog* are also detected in the above-mentioned stem cells [22,23,24,25]. Additionally, *Pou5f1* and *Nanog* participate in the differentiation and development of germ cells [20,21]. In mouse, Nanog serves as an epigenetic modifier involved in the maturation of haploid male germ cells [26], and the downregulation of *Pou5f1* results in the developmental arrest of oocytes [27]. Intriguingly, Nanog and Pou5f1 are significantly expressed in spermatocytes and spermatids, rather than spermatogonia in pig (*Sus scrofa*) testis [28]. In medaka, Nanog can regulate the expression level of *Cxcr4b* which is the guider of PGCs migration [29]. In gonads of medaka, zebrafish, and Japanese flounder, *Pou5f1* and *Nanog* are restricted to oogonia, oocytes, and spermatogonia [24,25,30,31]. Nevertheless, in large yellow croaker (*Larimichthys crocea*), *Pou5f1* is expressed in spermatogonia and primary spermatocytes, as well as all female germ cells [32]. In blunt-snout bream (*Megalobrama amblycephala*), *Nanog* is detected in all male germ cells and most female germ cells [33]. In goldfish (*Carassius auratus*), the *Nanog* promoter is in a state of hypermethylation in oocytes, while hypomethylation in sperm [34]. The expressions of *Pou5f1* and *Nanog* in germ cells indicate that they are functionally important in sustaining germ cell development and gametogenesis in mammals and fish.

Orange-spotted grouper (*E. coioides*) is a protogynous hermaphroditic fish whose gonad first develops as matured ovary and then reverses into testis, and therefore is regarded as an ideal model for studying the mechanism of sex reversal [35]. *Pou5f1* and *Nanog* have been characterized as germ cell-specific genes in some dioecious fish, but their expression patterns present a species difference to a variable extent [24,25,30,31,32,33]. In this study, *Pou5f1* (*EcPou5f1*) and *Nanog* (*EcNanog*) were cloned and characterized in orange-spotted grouper, and their expression patterns were analyzed during gametogenesis. These results would contribute to further studies on the mechanisms underlying PGCs formation and migration, germ cell development, and sex reversal in hermaphroditic fish.

## 2. Materials and Methods

### 2.1. Fish and Ethics

Animal experiments in this study were performed under the guidelines and approval of the Institutional Animal Care and use Committee of Sun Yat-Sen University. Female orange-spotted groupers with 25–35 cm in body length and 0.5–1 kg in body weight were bought from Huangsha Fisheries Trading Market, Guangzhou, Guangdong, China. Male orange-spotted groupers with 60–70 cm in body length and 5 kg in body weight were bought from Marine Fisheries Development Center of Guangdong Province, Huizhou, Guangdong, China. Groupers were anesthetized euthanized with 30 mg/L eugenol (Solarbio, Beijing, China) before being sacrificed. Tissue samples were as follows: brain, pituitary, gill filament, heart, head kidney, kidney, muscle, liver, stomach, intestine, spleen, testis, and ovary. These tissues were collected from groupers for the subsequent experiments, including RNA extraction, protein extraction, and frozen section.

### 2.2. Cloning of Full-Length cDNA Sequences

Full-length cDNA sequences of *EcPou5f1* and *EcNanog* were cloned from testis using SMARTer RACE cDNA Amplification Kit (Clontech Laboratories Inc., Mountain View, CA, USA). Based on the cDNA fragments of *EcPou5f1* and *EcNanog* in *E. coioides* genome data (unpublished data), specific primers were designed and listed in Table 1. Two rounds of PCR were carried out for amplifying the 5′ and 3′ cDNA ends. Pou5f1-F1 and UPM comprising UPM long and UPM short were mixed for the first round 3′ RACE amplification, while Pou5f1-R1 and UPM were employed for the first round 5′ RACE amplification. Pou5f1-R2 and NUP, as well as Pou5f1-F2 and NUP, were used for the second round 5′ and 3′ RACE amplification, respectively. The 5′ and 3′ cDNA end sequences of EcNanog were obtained via the same method. PCR procedure was as follows: denaturation at 95 °C for 1 min, followed by 35 cycles at 95 °C for 10 s, 58 °C for 10 s, 72 °C for 1 min, and a final extension of 5 min at 72 °C After agarose gel electrophoresis, the desired bands were purified by Gel Extraction Kit (Omega Bio-Tek, Norcross, GA, USA) and then sequenced by Sangon Biotech Company (Shanghai, China). According to sequencing data of purified DNA products, the 3′ and 5′ cDNA sequences of *EcPou5f1* and *EcNanog* were spliced into full-length cDNA sequences. Amino acid sequences of EcPou5f1 and EcNanog were predicted and performed for multiple sequence alignments using DNAMAN version 7.0 (Lynnon Biosoft, Quebec, QC, Canada). Phylogenetic trees were generated with MEGA version 5.0 (Biodesign Institute, Tempe, AZ, USA).

### 2.3. Total RNA Extraction and PCR

Total RNA of tissue was extracted with TRIzol reagent (Invitrogen, Carlsbad, CA, USA). RNA quality was evaluated by agarose gel electrophoresis. The cDNA was synthesized with 1 µg total RNA using ReverTra Ace qPCR RT Master Mix with gDNA Remover (Toyobo, Osaka, Japan). Real-time quantitative PCR (RT-qPCR) was performed on a Roche LightCycler 480 System (Roche Diagnostics, San Francisco, CA, USA) with SYBR Green Realtime PCR Master Mix (Toyobo). Produce was as follows: 30 s at 95 °C, followed by 40 cycles of 5 s at 95 °C, 5 s at 58 °C, and 15 s at 72 °C, with final step for 15 s at 95 °C and 30 s at 60 °C. Semi-quantitative PCRs for about 240 bp DNA fragments were performed with Taq PCR StarMix (Genstar, Shanghai, China), and PCR procedure was as follows: initial denaturation at 94 °C for 2 min, followed by 35 cycles of 30 s at 94 °C, 30 s at 58 °C, and 15 s at 72 °C, finally 72 °C for 5 min. Semi-quantitative PCR for the 1299 bp open reading frame (ORF) of *EcNanog* was performed with KOD One PCR Master Mix-Blue (Toyobo), and PCR procedure was as follows: initial denaturation at 94 °C for 2 min, followed by 35 cycles of 5 s at 98 °C, 5 s at 60 °C, and 5 s at 68 °C, finally 68 °C for 5 min. After gel electrophoresis, bands were photographed with a Tanon 1600 image system (Tanon, Shanghai, China). The *β-Actin* was used as an internal control. Genetic expression quantifications were normalized to *β-Actin*. Primers were listed in Table 1.

### 2.4. Western Blotting

The monoclonal anti-Nanog antibody from medaka was provided by Professor Hongyan Xu [36]. The total protein of tissues was extracted with RIPA Lysis Buffer (Beyotime, Shanghai, China) and mixed with SDS-PAGE Sample Loading Buffer (Beyotime). After being boiled for 5 min, 10 µL protein buffer was loaded into a lane, electrophoresed through 10% SDS-PAGE gels, and electroblotted onto polyvinylidene difluoride membrane (Merck Millipore, Billerica, MA, USA) by an electroblotter (BioRad, Hercules, CA, USA). The membrane was blocked with 5% BSA (Solarbio) for 1 h. After being washed with TBS (Solarbio), the membrane was incubated with anti-β-Actin antibody (Bioss, Beijing, China) or anti-Nanog antibody (1:1000 dilution in TBS) at 4 °C overnight. Then, the membrane was washed with TBS and incubated with HRP-conjugated goat anti-rabbit IgG (Bioss) (1:2000 dilution in TBS) for 2 h. Finally, protein blots were colored with Chemiluminescent Substrate for Western blotting Kit (Cyanagen, Bologna, Italy) and imaged by an Alliance MINI HD9 system (Uvitec, Cambridge, UK). The β-Actin was used as an internal control.

### 2.5. In Situ Hybridization (ISH)

ISH protocol was described in our previous study [37]. Briefly, after being fixed in 4% paraformaldehyde (Sangon, Shanghai, China) at 4 °C overnight, gonads were dehydrated with gradient methanol/PBS from 20% to 100% methanol, and then stored at −20 °C until further use. Gonads were rehydrated with gradient methanol/PBS up to 100% PBS, immersed in 30% (*w*/*v*) sucrose at 4 °C overnight, and embedded in OCT compound (SAKURA Tissue-Tek, Atlanta, GA, USA). These gonad samples were cryosectioned at 4 µm using a Leica RM2135 Microtome (Leica, Wetzlar, Germany). Probes were synthesized using DIG RNA labeling kit (Roche, Mannheim, Germany). Lengths of *EcPou5f1* and *EcNanog* probes were 1037 bp and 1299 bp, respectively. Ingredients of hybridization buffer were as follows: 50% deionized formamide, 5 × saline sodium citrate (SSC), 0.5 mg/mL salmon sperm RNA, 1 × Denhart’s solution, and 5% dextran sulphate. Gonadal sections were pre-hybridized with hybridization buffer for 2 h and then hybridized with 1 µg/mL DIG probes at 65 °C for 15 h. Subsequently, sections were washed with SSC and blocked with Blocking Reagent (Roche) for at least 1 h. Sections were incubated with anti-Digoxigenin-AP (Roche), then colored with NBT/BCIP (Roche). Photographs were imaged by a Leica DMI8 microscope (Leica).

### 2.6. Dual-Label ISH

Dual-label ISH protocol referred to our previous study [38]. The *Vasa* probe was 1040 bp and synthesized using Fluorescein RNA labeling kit (Roche). Gonadal sections were pre-hybridized for 2 h, then hybridized by 1 µg/mL *Vasa* probe and one of *EcPou5f1* and *EcNanog* probes at the same time at 65 °C for 15 h. Subsequently, sections were washed with SSC and blocked with Blocking Reagent for at least 1 h. The *Vasa* probe was incubated with anti-Fluorescein-POD (Roche), then stained red fluorescence for 10 min with TSA^TM^ PLUS Fluorescein system (PerkinElmer, Shelton, USA). After being washed, the *EcPou5f1* or *EcNanog* probes were incubated with anti-Digoxigenin-POD (Roche) and were stained green fluorescence for 10 min. Finally, gonadal sections were counterstained by DAPI (Solarbio) for cell nuclei staining. Sections were imaged with a Zeiss LSM 800 microscope (Zeiss, Jena, Germany) or a Leica TCS SP5 microscope (Leica).

### 2.7. Fluorescent Immunostaining

Gonadal sections were dried in a drying oven and washed with PBS. After being blocked with 5% goat serum (Sangon) for 1 h, sections were incubated with the anti-Nanog antibody for 2 h (1:200 dilution in PBS containing 2% goat serum). After being washed with PBS, sections were incubated with HRP-conjugated goat anti-rabbit IgG (1:3000 dilution in PBS) for 1 h. Signals were developed using the TSA^TM^ Plus Fluorescence System. The nucleus was colored by PI (Solarbio). Photographs were imaged by a Leica DMI8 microscope.

### 2.8. Statistical Analysis

All data were shown as the mean values ± SEM. Statistical analysis was implemented by one-way ANOVA and Student’s *t*-test. A probability level less than 0.05 (*p* < 0.05) was considered statistically significant. All statistics were carried out using GraphPad Prism version 5.0 (GraphPad Software, San Diego, CA, USA).

## 3. Results

### 3.1. Identifications of EcPou5f1 and EcNanog

**Figure 1:**
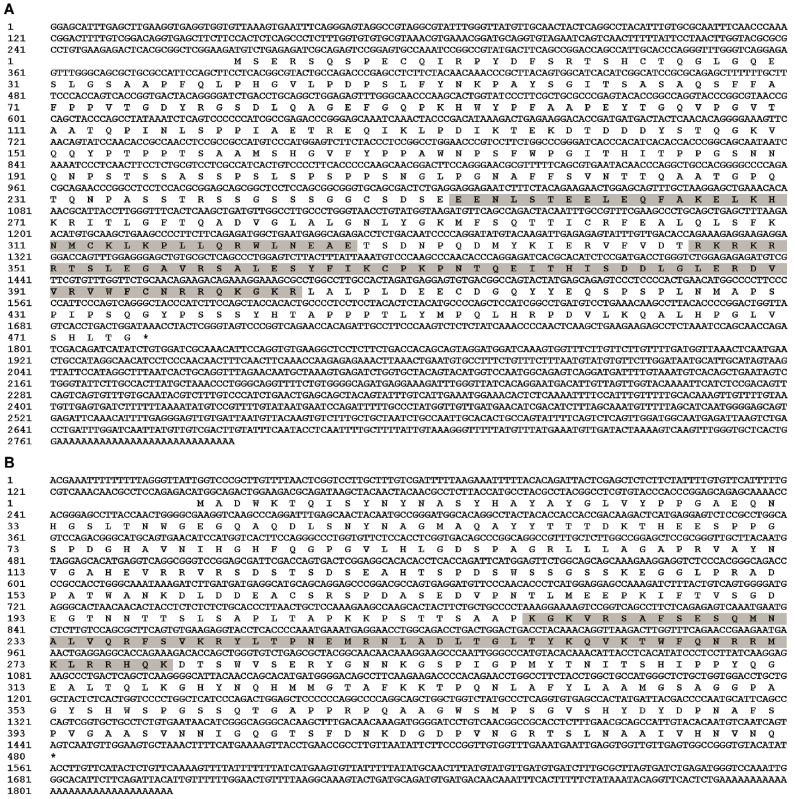
Full-length cDNA sequences and predicted polypeptides of (**A**) *EcPou5f1* and (**B**) *EcNanog*. Gray shadow: POUs and POUhD in (**A**), and HD in (**B**). Stop codon is labeled with an asterisk (*).

Full-length cDNA of *EcPou5f1* was 2790 bp with a 1428 bp ORF, a 269 bp 5′-untranslated region (UTR), and a 1093 bp 3′-UTR, as well as encoded a predicted protein of 475 amino acids containing POUs and POUhD (Figure 1A). Full-length cDNA of *EcNanog* was 1820 bp with a 1299 bp ORF encoding a 432 amino acid peptide with an HD domain, a 143 bp 5′-UTR, and a 378 bp 3′-UTR (Figure 1B). Sequences of *EcPou5f1* and *EcNanog* were deposited in GenBank with accession numbers OL439940 and OK415852, respectively.

**Figure 2:**
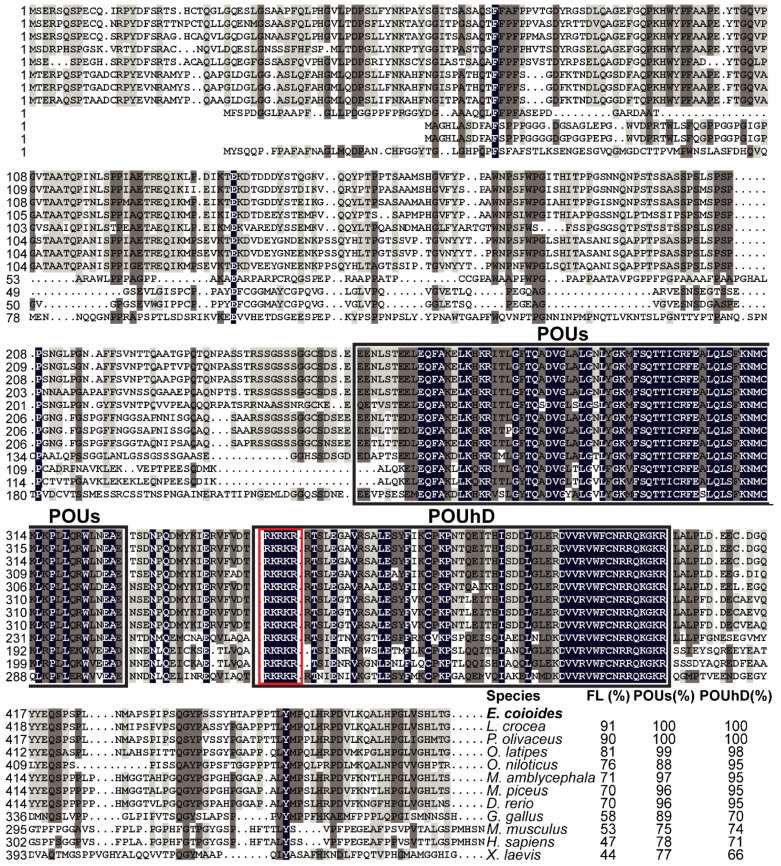
Multiple sequence alignment and comparison of EcPou5f1 with the homologs from other vertebrates. Black shadow: 100% identity; Brown shadow: 75% ≤ identity < 100%; Light brown shadow: 50% ≤ identity < 75%. Large black box: POUs and POUhD domains; Red box: a nuclear localization motif of RKRKR. The percent identity among EcPou5f1 and other Pou5f1 homologs in full-length (FL) and POU domains is displayed at the end of the alignment. The species’ name with a bold font indicates the EcPou5f1 protein of orange-spotted grouper. GenBank accession numbers for Pou5f1 are as follows: *E. coioides*, OL439940; *L. crocea*, NP_001290294; *P. olivaceus*, ALA23412; *O. latipes*, NP_001098339; *Oreochromis niloticus*, XP_003444455; *M. amblycephala*, AVV48156; *Mylopharyngodon piceus*, ATP62009; *D. rerio*, NP_571187; *Gallus gallus*, NP_001296301; *M. musculus*, NP_038661; *H. sapiens*, NP_002692; and *Xenopus laevis*, NP_001079832.

Multiple sequence alignments showed that EcPou5f1 protein had the conserved POUs and POUhD, as well as a nuclear localization motif of RKRKR (Figure 2). Full-length polypeptide of EcPou5f1 showed very high homology to fish Pou5f1 homologs from 70% to 91%, while low similar to tetrapod Pou5f1 homologs from 44% to 58% (Figure 2). In addition, POU domains showed a conserved feature in diverse vertebrates, with an identity ranging from 66% to 100% (Figure 2).

**Figure 3:**
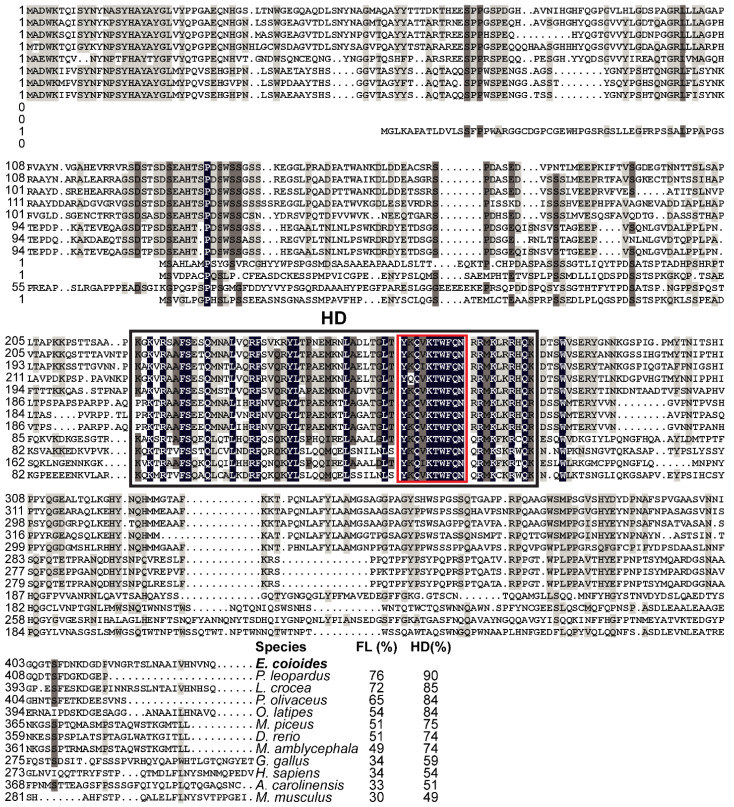
Multiple sequence alignment and comparison of EcNanog with the homologs from other vertebrates. Black shadow: 100% identity; Brown shadow: 75% ≤ identity < 100%; Light brown shadow: 50% ≤ identity < 75%. Large black box: HD domain; Red box: a nuclear localization motif of YKQVKTWFQN. The percent identity among EcNanog and its homologs in full-length (FL) and HD domain is displayed at the end of the alignment. The species name with a bold font indicates the EcNanog protein of orange-spotted grouper. GenBank accession numbers for Nanog are as follow: *E. coioides*, OK415852; *Plectropomus leopardus*, XP_042366645; *L. crocea*, XP_010739920; *P. olivaceus*, AGX84982; *O. latipes*, NP_001153902; *M. piceus*, ATP62010; *D. rerio*, NP_001091862; *M. amblycephala*, AMY98422; *G. gallus*, NP_001139614; *H. sapiens*, NP_079141; *M. musculus*, NP_082292; and *Anolis carolinensis*, XP_003216891.

EcNanog protein had a conserved HD domain with a nuclear localization motif of YKQVKTWFQN (Figure 3). EcNanog shared a low identity with tetrapod Nanog homologs, ranging from 30% to 34%, while a high identity with fish Nanog homologs ranging from 49% to 76% (Figure 3). Likewise, the greatest similarity appeared on the most important functional domains, i.e., HD domain, ranging from 49% to 90% (Figure 3).

**Figure 4:**
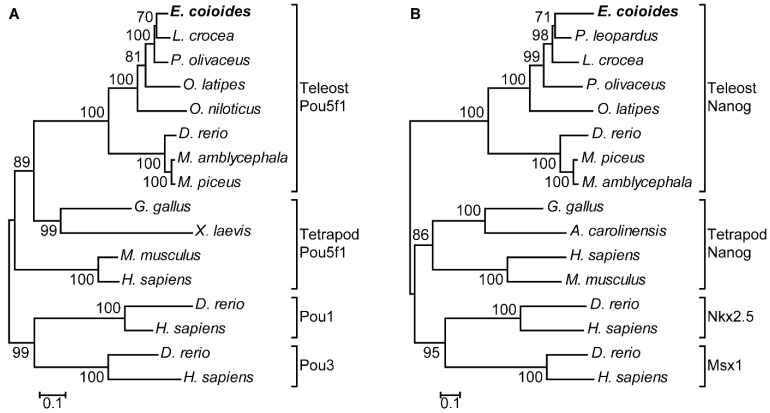
Phylogenetic trees of (**A**) EcPou5f1 and (**B**) EcNanog. Phylogenetic trees were deduced by MEGA version 5.0 using the neighbor-joining method with 1500 bootstrap replicates. Numerals at bifurcation points of phylogenetic tree are bootstrap values. The species names in the first line indicate the EcPou5f1 and EcNanog proteins of orange-spotted grouper. GenBank accession numbers for Pou5f1 are as follows: *E. coioides*, OL439940; *L. crocea*, NP_001290294; *P. olivaceus*, ALA23412; *O. latipes*, NP_001098339; *O. niloticus*, XP_003444455; *M. amblycephala*, AVV48156; *M. piceus*, ATP62009; *D. rerio*, NP_571187; *G. gallus*, NP_001296301; *M. musculus*, NP_038661; *H. sapiens*, NP_002692; and *X. laevis*, NP_001079832. GenBank accession numbers for Pou1 and Pou3 are as follows: *D. rerio*, NP_998016, NP_571236; *H. sapiens*, NP_000297, NP_002690. GenBank accession numbers for Nanog are as follows: *E. coioides*, OK415852; *P. leopardus*, XP_042366645; *L. crocea*, XP_010739920; *P. olivaceus*, AGX84982; *O. latipes*, NP_001153902; *M. piceus*, ATP62010; *D. rerio*, NP_001091862; *M. amblycephala*, AMY98422; *G. gallus*, NP_001139614; *H. sapiens*, NP_079141; *M. musculus*, NP_082292; *A. carolinensis*, XP_003216891. GenBank accession numbers for Nkx2.5 and Msx1 are as follow: *D. rerio*, NP_571496, NP_571348; and *H. sapiens*, NP_004378, NP_002439.

In phylogenetic tree analysis, EcPou5f1 and EcNanog were respectively clustered into a single clade with fish homologs and separated from other POU and HD proteins, including Pou1, Pou3, Nkx2.5, and Msx1 (Figure 4A,B).

### 3.2. Tissue Distributions of EcPou5f1 and EcNanog

**Figure 5:**
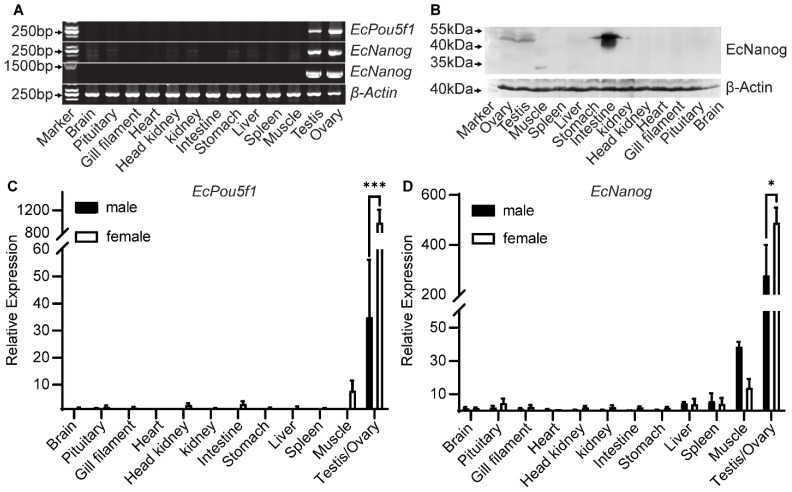
Expression pattern analyses of *EcPou5f1* and *EcNanog*. (**A**) Semi-quantitative PCR analyses of *EcPou5f1* and *EcNanog* in 13 tissues. The sizes of DNA fragments: *EcPou5f1*, 245 bp; *EcNanog*, 240 bp, 1299 bp; *β-Actin*, 235 bp. (**B**) Western blotting analysis of EcNanog protein in 13 tissues. (**C**,**D**) RT-qPCR analyses of *EcPou5f1* and *EcNanog* in 13 tissues. The data in **C** and **D** are presented as the mean ± SEM (*n* = 3). The values with asterisks are significantly different with *p* < 0.05 (*) or *p* < 0.001 (***). The *β-Actin* was used as an internal control.

Semi-quantitative PCR results showed that a 245 bp fragment of *EcPou5f1* was limited to gonads, whereas a 240 bp fragment of *EcNanog* was highly detected in gonads and weakly in other tissues, including brain, pituitary, head kidney, kidney, stomach, liver, and muscle (Figure 5A). However, the 1299 bp ORF of *EcNanog* was only amplified in gonads (Figure 5A). In Western blotting, EcNanog signals were detected as three strong bands between 40 and 55 kDa in intestine, a distinct band between 40 and 55 kDa in gonads and stomach, a single band about 35 kDa in muscle, a weak band between 40 and 55 kDa in kidney, as wells as no bands in other somatic tissues (Figure 5B). In RT-qPCR analyses, *EcPou5f1* and *EcNanog* were extremely expressed in gonads with a higher level in ovary than in testis (Figure 5C,D).

### 3.3. Chemical ISH of EcPou5f1 and EcNanog in Gonads

**Figure 6:**
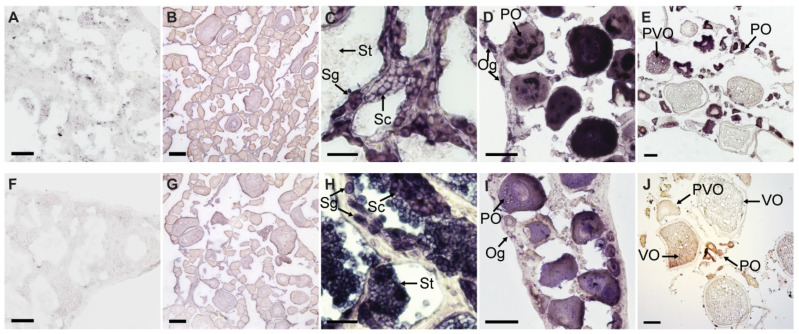
Chemical ISH of *EcPou5f1* and *EcNanog* in gonads of orange-spotted grouper. (**A**,**B**) Sense probe signal of *EcPou5f1* in testis and ovary. (**C**–**E**) Antisense probe signal of *EcPou5f1* in testis and ovaries. (**F**,**G**) Sense probe signal of *EcNanog* in testis and ovary. (**H**–**J**) Antisense probe signal of *EcNanog* in testis and ovaries. Sg, Spermatogonium; Sc, Spermatocyte; St, spermatid; Og, oogonium; PO, primary growth stage oocyte; PVO, cortical-alveolus stage oocyte; VO, vitellogenic stage oocyte. Scale bars: 50 µm in (**A**,**D**,**F**,**I**); 100 µm in (**B**,**E**,**G**,**J**); 20 µm in (**C**,**H**).

The sense riboprobes of *EcPou5f1* and *EcNanog* showed no specific signal in ovary and testis (Figure 6A,B,F,G). In testis, the antisense probe signal of *EcPou5f1* was intense in spermatogonia, moderate in spermatocytes, and no signal could be detected in spermatids (Figure 6C). In ovary, the antisense probe signal of *EcPou5f1* was obviously observed in oogonia, primary growth stage oocytes, and cortical-alveolus stage oocytes, but scarcely detected in vitellogenic stage oocytes (Figure 6D,E). In some primary growth stage oocytes, *EcPou5f1* mRNA signals were unevenly distributed in perinuclear speckles and nuclei (Figure 6D). The distribution of *EcNanog* mRNA in gonads had some differences from *EcPou5f1* mRNA. In testis, *EcNanog* mRNA signal was observed in the spermatogenic cells from spermatogonium to spermatid (Figure 6H). In ovary, *EcNanog* mRNA signal was detected in oogonia, primary growth stage oocytes, cortical-alveolus stage oocytes, and early vitellogenic stage oocytes, but scarcely in late vitellogenic stage oocytes (Figure 6I,J). In addition, it was observed that a few small oval cells, possibly oogonia or oogonial stem cells, were *EcPou5f1* or *EcNanog*-positive (Figure 6D,I).

### 3.4. Dual-Label ISH for EcPou5f1 or EcNanog with Vasa in Gonads

**Figure 7:**
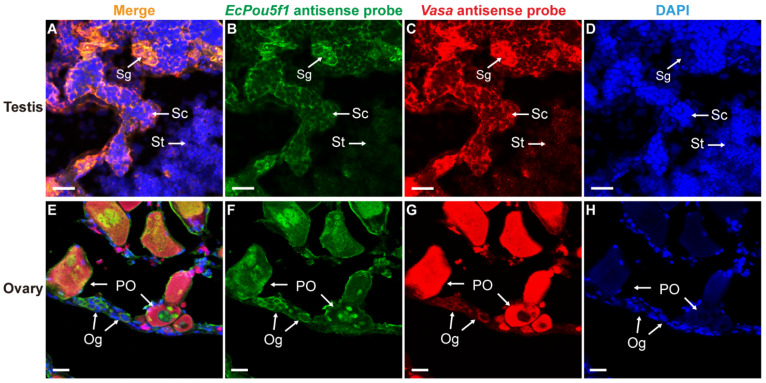
Co-localizations of *EcPou5f1* and *Vasa* in gonads of orange-spotted grouper. (**A**,**E**) Merged images. (**B**,**F**) Fluorescent signal of *EcPou5f1* antisense probe in testis and ovary. (**C**,**G**) Fluorescent signal of *Vasa* antisense probe in testis and ovary. (**D**,**H**) Nucleus was counterstained with DAPI. Sg, Spermatogonium; Sc, Spermatocyte; St, spermatid; Og, oogonium; PO, primary growth stage oocyte. Scale bars: 20 µm.

In order to further verify the reliability of *EcPou5f1* and *EcNanog* as germ cell-specific genes, we adopted *Vasa*, a well-known germ cell-specific gene [9], as a positive control in dual-label ISH. In testis and ovary, the mRNA signal of *EcPou5f1* had an identical localization with *Vasa* mRNA signal in male and female germ cells (Figure 7). It was worth mentioning that the very faInt. fluorescence of *EcPou5f1* mRNA could be observed in spermatids by Tyramide Signal Amplification system (Figure 7B). Similar to the chemical ISH result of *EcPou5f1* mRNA in ovary, the fluorescent signal of *EcPou5f1* mRNA was obviously observed in the cytoplasm of oogonia, and the perinuclear speckles and nuclei of some primary growth stage oocytes (Figure 7F). In ovary, the small oval cells, possibly oogonia or oogonial stem cells, with *EcPou5f1* signal would be marked by *Vasa* signal (Figure 7E–G).

**Figure 8:**
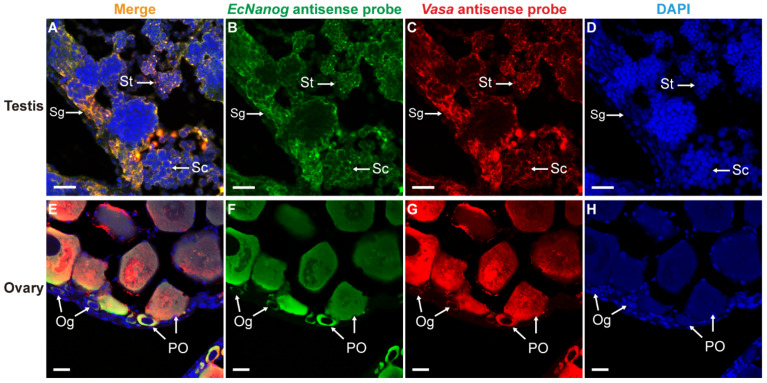
Co-localizations of *EcNanog* and *Vasa* in gonads of orange-spotted grouper. (**A**,**E**) Merged images. (**B**,**F**) Fluorescent signal of *EcNanog* antisense probe in testis and ovary. (**C**,**G**) Fluorescent signal of *Vasa* antisense probe in testis and ovary. (**D**,**H**) Nucleus was counterstained with DAPI. Sg, Spermatogonium; Sc, Spermatocyte; St, spermatid; Og, oogonium; PO, primary growth stage oocyte. Scale bars: 20 µm.

Likewise, *EcNanog* signal had an identical localization with *Vasa* signal in male and female germ cells (Figure 8). In testis, the *EcNanog* and *Vasa* signals were detected in different spermatogenic cells (Figure 8B,C). The fluorescent signals of *EcNanog* and *Vasa* were distributed in the whole cytoplasm of female germ cells (Figure 8F,G). In ovary, the small oval cells, possibly oogonia or oogonial stem cells, showed the *EcNanog* and *Vasa*-positive signals at the same time (Figure 8E–G).

### 3.5. Immunohistofluorescence of Anti-Nanog Antibody in Gonads

**Figure 9:**
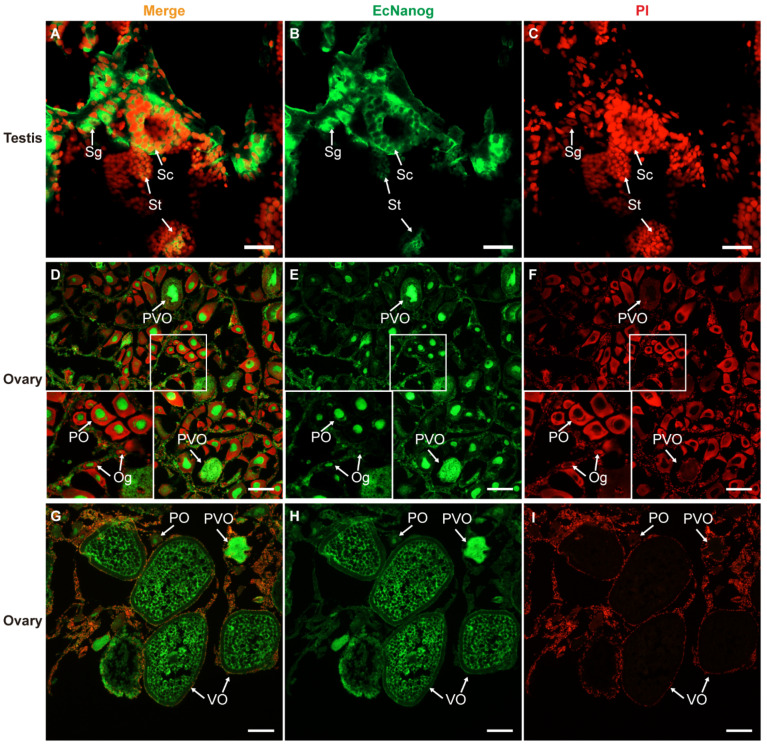
Immunohistofluorescence staining for anti-Nanog antibody in gonads of orange-spotted grouper. (**A**,**D**,**G**) Merged images. (**B**,**E**,**H**) Fluorescent signal of anti-Nanog antibody in testis and ovaries. (**C**,**F**,**I**) Nucleus was counterstained with PI. Sg, Spermatogonium; Sc, Spermatocyte; St, spermatid; Og, oogonium; PO, primary growth stage oocyte; PVO, the cortical-alveolus stage oocyte; VO, vitellogenic stage oocyte. Scale bars: 20 µm in (**A**–**C**); 100 µm in (**D**–**I**).

Immunohistofluorescence was carried out for examining the localization of EcNanog protein in gonads of orange-spotted grouper. In testis, the signal of anti-Nanog antibody was mainly detected in the nucleus and cytoplasm of spermatogonia and the cytoplasm of spermatocytes, while slight in spermatids (Figure 9A–C). In ovary, the signal of anti-Nanog antibody was predominantly detected in the nuclei of oogonia and primary growth stage oocytes, and then gradually spread in the cytoplasm of cortical alveolus stage oocytes, up to a homogeneous distribution in the whole vitellogenic stage oocytes (Figure 9D–I).

## 4. Discussion

Pluripotency markers Pou5f1 and Nanog are generally regarded as core transcription factors sustaining stem cell pluripotency and embryogenesis [11,12,13]. However, their roles are unclear in germ cell development and gametogenesis in teleost fish. In this study, the cDNA sequences and expression patterns of *EcPou5f1* and *EcNanog* were characterized and analyzed in a protogynous hermaphroditic fish, orange-spotted grouper. Sequence analysis showed that EcPou5f1 protein possessed a conserved POU domain, and EcNanog protein had a conserved HD domain. The similarity of POU domain and HD domain is much higher than the full-length sequences in various species. This situation reflects the conserved nature of Pou5f1 and Nanog in the process of evolution to a certain extent. For instance, the Nanog required for inducing mouse IPSCs can be replaced with chicken or zebrafish Nanog [39], and other tetrapod Pou5f1 can maintain the pluripotency and self-renewal of mouse ESCs [40,41]. Additionally, sequence alignments revealed that EcPou5f1 and EcNanog shared the highest homology with the orthologs in fish, in agreement with phylogenetic tree analysis. The greatest homology among fish implies that the functions of EcPou5f1 and EcNanog in orange-spotted grouper would be very similar to other fish homologs.

*EcPou5f1* and *EcNanog* show two discrepant tissue distribution patterns. The protein and short DNA fragment of *EcNanog* were detected in gonads and some nongonadal tissues, e.g., stomach and muscle, whereas the ORF region of *EcNanog* was only detected in gonads. This is consistent with the PCR result of *Nanog* in blunt-snout bream, the short fragment is detected in gonads and nongonadal tissues, but the long fragment just exists in gonads [33]. In zebrafish, the transcript and protein of *Nanog* are distinctly detected in gonads, liver, and heart [42]. On the contrary, in adult Japanese flounder, *Nanog* is restrictively expressed in ovary and testis [25]. We deduce that the alternative splicing of *EcNanog* lead to the inconsistent PCR amplification between the short and long fragments, and the appearance of protein bands in some somatic tissues that may possess Nanog-positive stem cells [33,43,44]. Similar to the tissue distribution pattern of Japanese flounder *Pou5f1* [30], *EcPou5f1* was restrictively expressed in gonads. Nonetheless, some somatic tissues of medaka and Chinese sturgeon (*Acipenser sinensis*), for example, brain, can also express *Pou5f1* [22,45]. Pou5f1 protein is a direct regulator of *Nanog* transcript [18]. Intriguingly, *EcPou5f1* was not detected in those nongonadal tissues expressing the short DNA fragment or protein of *EcNanog*. We guess that other regulators, such as Sox2 and Klf4, and the auto-regulatory of Nanog might account for the absence of *EcPou5f1* in nongonadal tissues [46,47]. In short, the tissue distributions of *Pou5f1* and *Nanog* show species differences to a variable extent in diverse fish, and suggest that they play multifunctional roles in gonads and somatic tissues.

*EcPou5f1* and *EcNanog* are dependable germ cell-specific genes in orange-spotted grouper. *EcPou5f1* was restricted to germ cells, but scarce in vitellogenic stage oocytes. Using Tyramide Signal Amplification system, *EcPou5f1* signal was faintly observed in spermatids, suggesting that a few *EcPou5f1* transcripts existed in spermatids. These ISH results indicate that *EcPou5f1* exists exclusively in germ cells of gonads and would downregulate dramatically from spermatocyte to spermatid. In medaka and Japanese flounder, *Pou5f1* is limited to spermatogonia, oogonia, and most oocytes, but absent in spermatocytes and spermatids [22,30]. In testis of Nile tilapia, Pou5f1 protein shows a specific localization in undifferentiated spermatogonia [48,49]. However, in large yellow croaker, *Pou5f1* is detected in spermatogonia and primary spermatocytes [32]. *EcNanog* was detected in all male germ cells and the female germ cells from oogonia to early vitellogenic stage oocytes. A similar expression pattern is also observed for *Nanog* in gonads of blunt-snout bream [33]. However, in testes of zebrafish, medaka, and Japanese flounder, *Nanog* is only expressed in spermatogonia [25,31,42]. The differential expression in differentiated male germ cells implies that *EcPou5f1* and *EcNanog* may participate in spermatogenesis, whereas the *Pou5f1* and/or *Nanog* of medaka, zebrafish, Japanese flounder, and Nile tilapia is mainly responsible for the pluripotency and self-renewal of spermatogonia. In accordance with the *EcNanog* ISH, EcNanog protein specifically existed in all male and female germ cells, including vitellogenic stage oocyte in which *EcNanog* mRNA was difficultly detected. EcNanog contained a motif of YKQVKTWFQN that had been identified as a nuclear localization motif in human Nanog [50]. As expected, fluorescent immunostaining revealed the nuclear localization of EcNanog in spermatogonia, oogonia, and primary growth stage oocytes. This is also supported by the nuclear localization of Nanog in zebrafish and blunt-snout bream [33,42]. Interestingly, we observed that EcNanog also existed in the cytoplasm of spermatocytes and spermatids. A similar localization of Nanog is observed in the spermatocytes and spermatids of pig testis [28]. The translocation of Nanog from nucleus to cytoplasm may imply the loss of function sustaining the pluripotency characteristics of spermatogonia. Although the subcellular localization of EcPou5f1 protein was not tested in this study, we speculated that EcPou5f1 might also locate in the nuclei of germ stem cells, on account of the nuclear localization signal RKRKR [50]. *Vasa* is a widely accepted germline-specific marker [51,52], and shows a specific expression in the germ cell lineage in many animals, e.g., medaka [53] and mouse [54]. Our previous study has demonstrated that *Vasa* is exclusively expressed in germ cells in gonads of orange-spotted grouper [9]. In adult testis of rhesus monkey (*Macaca mulatta*), Nanog is restrictively expressed in all spermatogenic cells, most of which are Vasa-positive [55]. In human fetal testis and ovary, Pou5f1 is limited to some fetal germ cells, whereas only a part of the cell shows the low intensity immunofluorescences of Pou5f1 and Vasa [20]. In this study, dual-label ISH analysis revealed that *Vasa* was co-localized with *EcPou5f1* and *EcNanog* in gonads. Furthermore, in ovary, the small oval cells expressing *EcPou5f1* or *EcNanog* would be marked by *Vasa* signal, suggesting that these cells exactly were oogonia or oogonial stem cells. On the basis of these results, we conclude that *EcPou5f1* and *EcNanog* are reliable germ cell-specific markers in orange-spotted grouper.

Although there have been a few studies about the functions of *Pou5f1* and *Nanog* in differentiated germ cells in mammals [26,27,28], it is widely accepted that *Pou5f1* and *Nanog* are mainly responsible for the pluripotency of germline stem cells [20,21,56,57]. Conversely, the teleost *Nanog* and *Pou5f1* are expressed in spermatogonia, oogonia, and a large proportion of oocytes, as well as have different expression patterns in the differentiated male germ cells in diverse fish [22,25,30,31,33,42,49]. Likewise, *EcPou5f1* and *EcNanog* can be detected in all spermatogonia and oogonia, most oocytes, spermatocytes, and spermatid. The high expressions of teleost *Nanog* and *Pou5f1* during oocyte development may be related to maternal inheritance, i.e., mRNA or protein delivery to the next generation [24,25,33]. The comparisons in differentiated male germ cells indicate that *Pou5f1* and *Nanog* may have some differences in promoting spermatogenesis in different fish. Moreover, the differentials among diverse fish would provide a new perspective for understanding the evolution and conserved nature of teleost *Pou5f1* and *Nanog*.

## 5. Conclusions

In summary, *EcPou5f1* and *EcNanog* are germ cell-specific marker genes and play important roles in gametogenesis in a protogynous hermaphroditic fish, orange-spotted grouper. Our findings would contribute to further studies on the molecular mechanism underlying germ cell development, gametogenesis, and sex reversal in hermaphroditic fish.

## Figures and Tables

**Table 1:** Primers for synthesizing RNA probes, cloning the full-length cDNAs, and analyzing gene expression levels.

Primer	Sequence (5′ to 3′)	Purpose
Vasa-F	GAGCCTGAGACCATCATC ^1^	ISH
Vasa-R	AGGACTCTTCACACTGTTG ^1^	ISH
Pou5f1-F	TCTACAACAAACCCGCTTACAGT	ISH
Pou5f1-R	GCAGAACCAAACACGAACGAC	ISH
Nanog-F	ATGGCAGACTGGAAGACGCAGATAA	ISH, RT-PCR
Nanog-R	CTACTGATTGACATTGTGTACAATG	ISH, RT-PCR
Pou5f1-F1	TGCGTCCTCGCCATCACTG	RACE
Pou5f1-F2	AACGCATTACCTTGGGTTTCAC	RACE
Pou5f1-R1	GCTCCCTCCAAACTGGTCCTC	RACE
Pou5f1-R2	TGTTGAGTAGTCATCATCGGTGTCC	RACE
Nanog-F1	CTAACAACACTACCTCTC	RACE
Nanog-F2	GTGGACCTGCTGGCTACT	RACE
Nanog-R1	CTTGTCGGTGGTGGTGTA	RACE
Nanog-R2	TATCTGCGTCTTCCAGTCTGCCAT	RACE
UPM long	CTAATACGACTCACTATAGGGCAAGCAGTGGTATCAACGCAGAGT ^2^	RACE
UPM short	CTAATACGACTCACTATAGGGC ^2^	RACE
NUP	AAGCAGTGGTAACAACGCAGAGT ^2^	RACE
Pou5f1-F3	AACGCATTACCTTGGGTTTCACT	RT-PCR
Pou5f1-R3	GGTCCTCCTCTTCCTCTTTCTGG	RT-PCR
Nanog-F3	AACTGAGGAGGCACCAGAAAGAC	RT-PCR
Nanog-R3	CAGCAGGTCCACCAGCAGAG	RT-PCR
β-Actin-F	TTCACCACCACAGCCGAGA	RT-PCR
β-Actin-R	TGGTCTCGTGGATTCCGCAG	RT-PCR
Pou5f1-F4	TGTCCCAAGCCCAACACCCA	RT-qPCR
Pou5f1-R4	CCAGGCGCTTTCCTTTCTGTC	RT-qPCR
Nanog-F4	GGAGGCACCAGAAAGACACCA	RT-qPCR
Nanog-R4	CTGAGTCAGGGCTTCTCCTTGATA	RT-qPCR
β-Actin-F1	AAATCGCCGCACTGGTTGTT	RT-qPCR
β-Actin-R1	CCCTCTTGCTCTGGGCTTCAT	RT-qPCR

^1^ The primer sequences of *Vasa* were from Qu et al. [9]; ^2^ The primers were provided by SMARTer RACE cDNA Amplification Kit; ISH, in situ hybridization; RACE, Rapid Amplification of cDNA Ends; RT-PCR, Reverse Transcription PCR; RT-qPCR, Real-time quantitative PCR.

## Data Availability

The study did not report any other data.
